# Photonics-Based Multifunction System for Radar Signal Transmit-Receive Processing and Frequency Measurement

**DOI:** 10.3390/mi15091080

**Published:** 2024-08-27

**Authors:** Dengcai Yang, Ya Zhang, Feng Yang, Mei Yang, Yinhua Cao

**Affiliations:** School of Physics and Optoelectronic Engineering, Institute of Laser Engineering, Beijing University of Technology, Beijing 100124, China

**Keywords:** microwave photonics, multifunction system, de-chirp reception, self-interference cancellation, frequency measurement

## Abstract

A novel photonic-assisted multifunctional radar system was proposed and experimentally investigated. This system can simultaneously achieve frequency-doubled linear frequency modulation (LFM) signal generation, de-chirp reception, self-interference cancellation, and frequency measurement in an integrated transmit-receive radar. First, a high-frequency and broadband LO signal was obtained with photonic frequency doubling, which improved the center frequency and bandwidth of the radar detection system. Then, photonic-assisted interference cancellation was used to reduce the impact of interference signals in radar de-chirp reception. Finally, the microwave frequency measurement was achieved by establishing a mapping relationship between the envelope response time of the intermediate frequency (IF) electrical filter and the microwave frequency to be tested. Both theoretical and experimental investigations were performed. The results showed that an LFM signal with a frequency range of 12–18 GHz was obtained with photonic frequency doubling. Photonic-assisted self-interference cancellation reduced the impact of interference signals in radar de-chirp reception by more than 12.1 dB for an LFM signal bandwidth of 6 GHz. In the frequency measurement module, the difference between the frequency to be tested, generated by the external signal source, and that calculated in the experiment is the measurement error, and a measurement resolution better than 14 MHz was achieved in the range of 12.14 GHz–18.14 GHz. The proposed system is suitable for miniaturized multifunctional radar signal processing systems with continuous operation of transmitting and receiving antennas in unmanned aerial vehicles (UAVs), automotive radar, relatively close spatial locations, and so on. In addition, it can simplify the system structure and reduce space occupation.

## 1. Introduction

Microwave photonics (MWP) technology has become an effective way to overcome the bottleneck of traditional electronic technology and has a wide range of applications in radar, electronic warfare, and other electronic systems because of its large bandwidth, low transmission loss, and anti-electromagnetic interference [1,2,3,4,5]. In recent years, many studies have been conducted to reduce the cost and volume of hardware in MWP systems. One of the important research interests is to incorporate multiple different functions into a single system [6,7,8], which not only reduces the platform energy consumption, space occupation, and cost but also complements the functions of each other and fully utilizes the functions of each unit. In addition, the multifunctional MWP system has good dynamic organization capability, which can improve operational performance [1,9].

The frequency-modulated continuous wave (FMCW) radar with integrated transmit-receive is a typical multifunctional signal processing system. In particular, miniaturized and integrated FMCW radar includes the generation of frequency-modulated microwave signals [10,11] and de-chirp reception, which can reduce the data processing volume and improve detection accuracy. This may become the preferred solution for miniaturization platforms such as unmanned aerial vehicles (UAVs), automotive radar, and so on. In these miniaturized platforms, the transmitting and receiving antennas work continuously, their spatial positions are relatively close, and the transmitted signal directly leaks to the receiving antenna. This greatly reduces the sensitivity of the receiver and even saturates and is unable to work, which limits the dynamic range of miniaturized and integrated FMCW radar systems [12]. There are many studies related to eliminating self-interference that leaks from the transmitter or transmitting signal leakage [13,14,15,16,17,18,19,20]. Many self-interference cancellation methods have been proposed in the electric domain, including single-path self-interference cancellation methods [13,14] and multipath self-interference cancellation methods [15,16]. However, due to electronic bottlenecks, the operating bandwidth and operating frequency of these methods cannot meet the increasing requirements of modern radar systems. 

Moreover, several RF self-interference cancellation methods based on microwave photonics have also been proposed [17,18,19,20]. The basic principle of self-interference cancellation in these methods is to introduce a reference signal with adjustable amplitude and phase and adjust the reference signal through the optical domain to achieve interference cancellation by making it have the same amplitude and out-of-phase as the interference signal. Therefore, the correlation between the provided reference signal and the self-interference signal largely determines the cancellation performance of the system. To provide optical domain cancellation schemes, photonic-assisted self-interference cancellation may use devices such as dual-parallel Mach‒Zehnder modulators (DPMZMs) [17,18], dual-drive Mach‒Zehnder modulators (DDMZMs) [19] or dual-polarization binary phase-shift keying modulators (DP-BPSKs) [20] to load interference signals, reference signals, and cancellation adjustments.

On the other hand, microwave frequency measurement is another important task in electronic systems. Photonics-assisted microwave frequency measurements, including frequency-amplitude mapping [21,22], frequency-space mapping [23,24], and frequency-time mapping [25,26,27,28,29], have been proposed. The basic principle of frequency-amplitude mapping is to construct an amplitude comparison function (ACF), which is the ratio between two different optical or microwave power functions. Typically, frequency-power mapping is achieved mainly through dispersive devices [21] and interferometers [22]. However, it is difficult to achieve multitone signal measurements via frequency-amplitude mapping. The frequency-space mapping method was developed on the basis of channelized frequency measurements and can achieve a wider frequency measurement range than the electrical channelized frequency measurement method. Since the bandwidth of optical bandpass filters is much larger than that of electrical filters, the frequency measurement accuracy and resolution of the frequency-space mapping method are poor. Frequency-time mapping can be realized by dispersive medium [25] or stimulated Brillouin scattering (SBS) effects [26], or frequency-time mapping can be realized by optical sideband scanning methods [27,28,29]. In [27], the linear frequency modulation (LFM) signal was used as a swept signal to establish a frequency-time mapping relationship for instantaneous frequency measurements of multifrequency and broadband signals. In [28], an LD was driven by a sawtooth waveform to produce an optical linear frequency-modulated signal to realize single sideband swept signals. The reconfigurable optical bandpass filter (OBPF) and dual-polarization MZM (Dpol-MZM) are combined to generate different optical sidebands by adjusting the reconfigurable OBPF [29]. These methods are based on single-sideband modulation or OBPF, which is difficult to modulate. Moreover, the frequency measurement methods utilizing LFM signals are affected by the mirror frequency signal, which degrades the measurement accuracy and causes frequency measurement blurring.

In this paper, a multifunctional system that can simultaneously achieve frequency-doubled linear frequency modulation signal generation, de-chirp reception, self-interference cancellation, and frequency measurement in an integrated transmit-receive radar is proposed, and experimental verification is also conducted. First, the frequency-doubled LO signal is obtained in the optical domain, which improves the center frequency and bandwidth of the radar detection system. Second, interference cancellation based on photonic technology has been utilized to reduce the impact of interference signals in radar de-chirp reception. Finally, the microwave frequency measurement is established by mapping between the envelope response time of the intermediate frequency (IF) electrical filter and the tested microwave frequency. Two low-frequency electrical filters with different center frequencies are used, which are combined with data acquisition and processing to solve the frequency ambiguity problem in the scanning frequency measurement system. In addition, the shared doubled frequency LO signal between the frequency measurement system and the radar signal processing system also increases the operation frequency range and simplifies the system structure.

## 2. Principle

**Figure 1:**
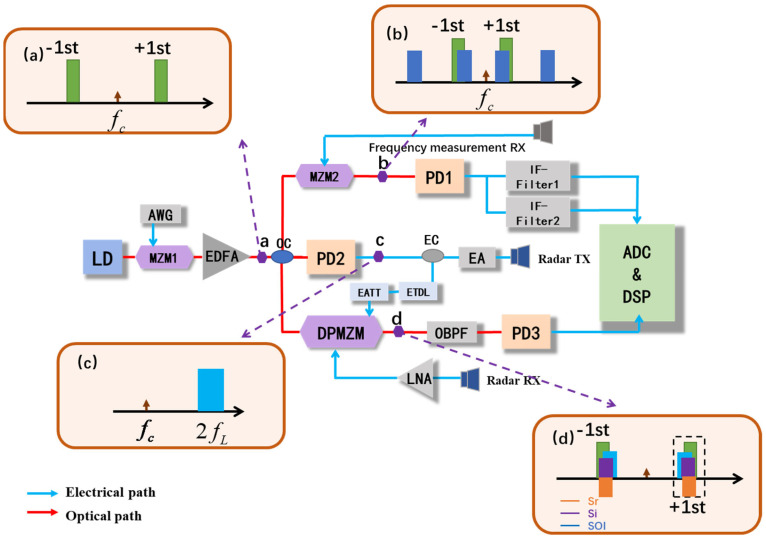
Schematic diagram of the proposed multifunctional microwave photonic system. The optical spectrum of (**a**) the CS-DSB and (**b**) the RF signal to be test, (**c**) the electrical spectrum of the frequency-doubled LFM signal after PD2, (**d**) the optical spectrum of self-interference cancellation. LD: laser diode; MZM: Mach–Zehnder modulator; DPMZM: dual-parallel Mach–Zehnder modulator; AWG: any waveform generator; EA: electrical amplifier; EATT: electrical amplitude tunable attenuator; ETDL: electrical time delay line; OBPF: optical bandpass filter; LNA: low noise amplifier; PD: photodetector.

Figure 1 shows a schematic diagram of the proposed photonic-based multifunctional system, which can simultaneously achieve frequency-doubled linear frequency modulation signal generation, de-chirp reception, self-interference cancellation, and frequency measurement in an integrated transmit-receive radar. The continuous wave (CW) light emitted from a laser diode (LD), which can be expressed as $E_{0}(t)=E_{0}\exp(j\omega_{c}t)$ is injected into a Mach–Zehnder modulator (MZM1). Here, $E_{0}$ and $\omega_{c}$ are the magnitude and angular frequency of the optical carrier, respectively, and $\omega_{c}=2\pi f_{c}$ and $f_{c}$ are the frequencies of the CW light. A linear frequency modulation signal generated by an arbitrary waveform generator (AWG) will be loaded as the local oscillator (LO) signal into MZM1, which biases at the minimum transmission point to obtain a carrier-suppressed double-sideband (CS-DSB) signal. The corresponding optical spectrum is shown in Figure 1a. The output of MZM1 can be written as

(1) $$E_{1}(t)\propto J_{1}(\alpha )\left\{\begin{array}{l} \exp\left[j2\pi (f_{c}+f_{LFM})t\right]\\ +\exp\left[j2\pi (f_{c}-f_{LFM})t\right] \end{array}\right\}$$

where $J_{1}(\cdot )$ represents the 1st-order Bessel function of the first kind and α is the modulation index of MZM1. $f_{LFM}=f_{0}+kt\;(0<t\leq T)$ is the instantaneous frequency of the LFM signal in a single period with T, and $f_{0}$ is the initial frequency of the LFM signal. The chirp rate *k* of the LFM can be written as k=B/T, where B represents the sweeping bandwidth of the LFM signal.

The generated CS-DSB signal is divided into two parts. One is sent as an optical carrier of the frequency measurement section to MZM2, which is driven by the signals under test (SUT) received from the antenna and operates at the quadrature point. The optical spectrum of MZM2 is shown in Figure 1b, and it can be expressed as

(2) $$\begin{array}{cc} E_{2}(t) & =E_{1}(t).\left\{J_{0}(\beta )+J_{1}(\beta )\exp\left(j2\pi f_{SUT}t\right)+J_{1}(\beta )\exp\left(-j2\pi f_{SUT}t\right)\right\}\\ & \propto J_{1}(\alpha )J_{1}(\beta )\left\{\begin{array}{l} \exp\left[j2\pi (f_{c}+f_{LFM}+f_{SUT})t\right]\\ +\exp\left[j2\pi (f_{c}-f_{LFM}+f_{SUT})t\right]\\ +\exp\left[j2\pi (f_{c}+f_{LFM}-f_{SUT})t\right]\\ +\exp\left[j2\pi (f_{c}-f_{LFM}-f_{SUT})t\right] \end{array}\right\}\\ & +J_{1}(\alpha )J_{0}(\beta )\left\{\begin{array}{l} \exp\left[j2\pi (f_{c}+f_{LFM})t\right]\\ +\exp\left[j2\pi (f_{c}-f_{LFM})t\right] \end{array}\right\} \end{array}$$

where β is the modulation index of MZM2 and $f_{SUT}$ is the frequency of the SUT. After MZM2, the optical signal is injected into a photodetector (PD1), and the output current of PD1 is denoted by

(3) $$\begin{array}{cc} i_{1}(t) & \propto dc+2{J^{2}}_{1}(\alpha )J_{0}(\beta )J_{1}(\beta )\cos[2\pi (f_{SUT}-2f_{LFM})t]\\ & +{J^{2}}_{1}(\alpha ){J^{2}}_{1}(\beta )\cos[2\pi (2f_{SUT}-2f_{LFM})t]\\ & +[{J^{2}}_{1}(\alpha ){J^{2}}_{0}(\beta )+2{J^{2}}_{1}(\alpha ){J^{2}}_{1}(\beta )]\cos\left(4\pi f_{LFM}t\right)\\ & +4{J^{2}}_{1}(\alpha )J_{1}(\beta )J_{0}(\beta )\cos(2\pi f_{SUT}t)\\ & +2{J^{2}}_{1}(\alpha ){J^{2}}_{1}(\beta )\cos(4\pi f_{SUT}t)\\ & +2{J^{2}}_{1}(\alpha )J_{0}(\beta )J_{1}(\beta )\cos[2\pi (f_{SUT}+2f_{LFM})t]\\ & +{J^{2}}_{1}(\alpha ){J^{2}}_{1}(\beta )\cos[2\pi (2f_{SUT}+2f_{LFM})t] \end{array}$$

As shown in Equation (3), the obtained signal contains a direct-current (dc) component and seven other frequency components. Due to the low-frequency response of PD1 in the proposed system, only the frequency component at $f_{SUT}-2f_{LFM}$ and the direct-current (dc) component are outputs. Then, a narrowband electrical IF filter is used to obtain the frequency component at $f_{1}$, which is the center frequency of the IF filter. The envelope signal of the IF signal can be expressed as

(4) $$\begin{array}{cc} e(t) & =A\delta (f_{1}-\left|2(f_{0}+kt)-f_{SUT}\right|)\\ & =\left\{\begin{array}{c} A,t=\frac{f_{1}-2f_{0}+f_{SUT}}{2k}\;or\;t=\frac{-f_{1}-2f_{0}+f_{SUT}}{2k},t\in [0,T]\\ 0,t\neq \frac{\pm f_{1}-2f_{0}+f_{SUT}}{2k},t\in [0,T] \end{array}\right. \end{array}$$

where A is the amplitude of the envelope signal. According to Equation (4), the output envelope signal of the IF filter is a periodic signal, and its period is the same as that of the LFM signal. Moreover, when the amplitude of the output envelope signal is nonzero, t is determined by $f_{SUT}$. Therefore, according to Equation (4), $f_{SUT}$ can be calculated by

(5) $$f_{SUT}=2f_{0}+2kt\pm f_{1}$$

**Figure 2:**
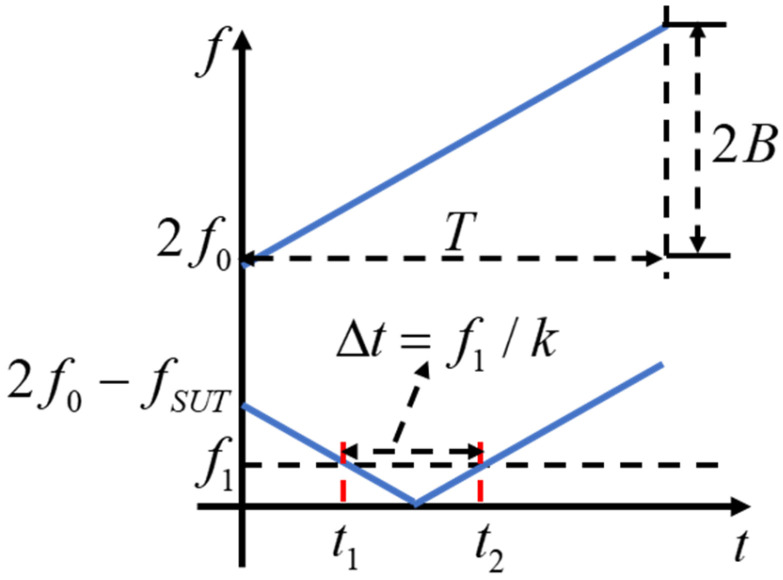
Principle of frequency-time mapping frequency measurement based on scanning frequency.

Owing to the periodic sawtooth scanning waveform of the LFM signal, the time pulse signal is generated when the difference in frequency between the LFM signal and the measured signal is equal to the center frequency of the IF filter. Therefore, to avoid frequency measurement crossing cycles, the actual frequency measurement bandwidth is the sweep bandwidth, but its starting frequency is related to the bandwidth of the IF filter and the starting frequency of the LFM signal, and the frequency measurement range is $2f_{0}+f_{1}$ to $2f_{0}+2kT+f_{1}$. The principle of time-frequency mapping frequency measurement based on scanning frequency is shown in Figure 2. From Equation (4) and Figure 2, it can be concluded that the signal under test corresponds to two time-pulses when the frequency measurement range is $2f_{0}+f_{1},\;2f_{0}+2kT-f_{1}$, and the time difference between the two time-pulses is fixed at $∆t=f_{1}/k$. It should be noted that this non-single mapping will lead to ambiguity in the frequency measurement. To avoid the measurement ambiguity caused by the image frequency relative to the SUT, the signal of the time pulse can be extracted with this fixed time interval of the time pulses from the time-frequency map. In the data acquisition and processing module, the front pulse appearing in pairs is taken, and the remainder is eliminated; then, the corresponding frequency of the time pulse is

(6) $$f_{SUT}=2kt_{1}+2f_{0}+f_{1}$$

When the frequency to be tested is greater than $2f_{0}+2kT-f_{1}$ and less than $2f_{0}+2kT$, the output of the IF filter has only one time-pulse, which is the front pulse. The corresponding time of the pulse is greater than $T-f_{1}/k$ and less than T. At this time, the calculated frequency is $f_{SUT}=2kt_{1}+2f_{0}+f_{1}$.

Calculating the frequency to be measured using the time corresponding to the peak value of the time pulse signal detected. Thus, the resolution of the frequency measurement is related to the temporal resolution ($∆t_{\min}$) of the time pulse signals. The resolution of the frequency measurement is

(7) $$f_{RES}=2k∆t_{\min}$$

On the one hand, the resolution of the frequency measurement depends on the number of sampling points of the time pulse signal by the ADC. By digitizing the ADC with a high-speed and highly effective number of bits, the impact of ADC sampling on the time pulse resolution can be reduced. On the other hand, owing to the bandwidth limitation of the IF filter, in actual testing systems, the time pulse signal is not an impulse function but rather a pulse envelope signal with a certain width. The full width at half maximum (FWHM) of the envelope signal is limited by the 3 dB bandwidth of the IF filter and the charging and discharging time of the IF filter (0.7/BIF) [30]. Therefore, the FWHM time of the time pulse signal is used to characterize the minimum time capability of the time pulse signals. The difference between the frequency to be tested, as generated by an external signal source, and the experimentally calculated frequency is the measurement error [29].

The proposed scheme can also be used for multifrequency microwave frequency measurements. In the data acquisition and processing module, first, the corresponding times of all pulses are collected; then, the time pulses are classified and sorted as mentioned above, and the frequency to be tested is calculated. However, when the time-frequency mapping of two microwave frequencies to be tested results in a time difference that is an integer multiple of the selected filter center frequency, the overlap of the previous frequency’s later time pulse and the next frequency’s earlier pulse interferes with the data processing, which can lead to missing the higher-frequency microwave frequencies. By adding another data acquisition and processing module with a tunable center frequency IF filter to determine whether there is pulse overlap in the fixed intermediate frequency channel, it is possible to distinguish between them and the time pulse signal obtained by tuning the center frequency. Tuning the center frequency of the IF filter, the number of time pulses measured at any center frequency is consistent with the number of time pulses in the fixed IF filter. At this time, the calculated frequency with the fixed center frequency IF filter is the actual measured frequency. If the number of time pulses measured by the two channels is inconsistent, there may be pulse overlap, which corresponds to the frequency to be tested by combining the time pulses of the two data acquisition and processing module channels. When two IF filters with different center frequencies are used, there is a difference in the fixed time difference ∆t for data processing in the two channels. Therefore, by fusing the number of time pulses and frequency measurement results of the channels of the fixed and tunable center frequency IF filter, multifrequency microwave signal frequency measurement values can be obtained. Furthermore, the LFM signal used in this system is tunable, and the range and speed of the frequency measurement can be changed by adjusting the bandwidth and duration of the LFM signal.

In the radar detection section, the optical signal from MZM1 is divided into two channels. In the upper channel, the optical signal is converted to electrical signals by PD2, and the spectrum of the generated signal is shown in Figure 1c. After that, one part of the generated electrical signal is sent to a DPMZM as a reference signal, and the other part is amplified by an electrical amplifier (EA) and sent to the transmitting antenna as the transmit signal. According to the characteristics of the LFM signal, the transmit signal can be represented as

(8) $$S_{T}(t)=A_{T}\cos\left(4\pi f_{0}t+4\pi kt^{2}\right)$$

where $A_{T}$ denotes the amplitude of the transmit frequency-doubled signal. Then, the corresponding echo signal $S_{SOI}(t)$ can be expressed as

(9) $$S_{SOI}(t)=A_{SOI}\cos\left[4\pi f_{0}t+4\pi k(t-\tau )t\right]$$

where $A_{SOI}$ is the amplitude of the echo signal and τ is the time delay between the echo signal and the transmit signal caused by the detecting target. The self-interference signals received from the receiving antenna $S_{L}(t)$ can be represented as

(10) $$S_{L}(t)=A_{L}\cos\left[4\pi f_{0}t+4\pi k(t-\tau^{\prime })t\right]$$

where $A_{L}$ is the amplitude of the echo signal and $\tau^{\prime }$ is the time delay due to direct transmission from the transmit-receive antenna. The reference signal $S_{R}(t)$, which is used to eliminate the self-interference signal $S_{R}(t)$, can be represented as

(11) $$S_{R}(t)=A_{R}\cos\left[4\pi f_{0}t+4\pi k(t-\tau^{\prime \prime })t\right]$$

where $A_{R}$ is the amplitude of the reference signal and $\tau^{\prime \prime }$ is the time delay caused by the electrical time delay line (ETDL).

In the lower channel, the CS-DSB signal is sent to a DPMZM as an optical carrier. By applying the received signal under interference and the reference signal on the two RF ports of the DPMZM, the sub-MZMs of the DPMZM are biased at the quadrature working point. To achieve reliable self-interference cancellation performance, an ETDL and an electrical amplitude tunable attenuator (EATT) are applied to the system to ensure consistency between the reference signal and the self-interference signal. Moreover, the main bias of the DPMZM is set toward the minimum number of transmission points. For the approximation of small signals, the high-order sidebands (n ≥ 2) are omitted. The output of the DPMZM can be expressed as

(12) $$\begin{array}{cc} E_{DPMZM}\left(t\right) & \propto \left[J_{0}(m_{1})+J_{0}(m_{2})-J_{0}(m_{3})\right]J_{1}(\alpha )\left\{\begin{array}{l} \exp\left[j2\pi (f_{c}+f_{0}+kt)t\right]\\ +\exp\left[j2\pi (f_{c}-f_{0}-kt)t\right] \end{array}\right\}\\ & +J_{1}(m_{1})J_{1}(\alpha )\left\{\begin{array}{l} \exp\left[j2\pi (f_{c}-f_{0}-kt+2k\tau )t\right]+\exp\left[j2\pi (f_{c}-3f_{0}-3kt+2k\tau )t\right]\\ +\exp\left[j2\pi (f_{c}+3f_{0}+3kt-2k\tau )t\right]+\exp\left[j2\pi (f_{c}+f_{0}+kt-2k\tau )t\right] \end{array}\right\}\\ & +J_{1}(m_{2})J_{1}(\alpha )\left\{\begin{array}{l} \exp\left[j2\pi (f_{c}-f_{0}-kt+2k\tau^{\prime })t\right]+\exp\left[j2\pi (f_{c}-3f_{0}-3kt+2k\tau^{\prime })t\right]\\ +\exp\left[j2\pi (f_{c}+3f_{0}+3kt-2k\tau^{\prime })t\right]+\exp\left[j2\pi (f_{c}+f_{0}+kt-2k\tau^{\prime })t\right] \end{array}\right\}\\ & -J_{1}(m_{3})J_{1}(\alpha )\left\{\begin{array}{l} \exp\left[j2\pi (f_{c}-f_{0}-kt+2k\tau^{\prime \prime })t\right]+\exp\left[j2\pi (f_{c}-3f_{0}-3kt+2k\tau^{\prime \prime })t\right]\\ +\exp\left[j2\pi (f_{c}+3f_{0}+3kt-2k\tau^{\prime \prime })t\right]+\exp\left[j2\pi (f_{c}+f_{0}+kt-2k\tau^{\prime \prime })t\right] \end{array}\right\} \end{array}$$

where $m_{1}$, $m_{2}$ and $m_{3}$ denote the modulation indices of the echo signal, self-interference signal, and reference signal in the sub-MZMs of the DPMZM, respectively. To eliminate self-interference signals from the received signal, Equation (12) shows that the amplitude and phase of the reference and interference signals should be well matched by adjusting the ETDL and EATT. When $J_{1}(m_{2})=J_{1}(m_{3})$ and $\tau^{\prime }=\tau^{\prime \prime }$, the effects of self-interference in the optical domain can be completely eliminated. Under the small-signal modulation condition, Equation (12) can be written as

(13) $$\begin{array}{cc} E_{DPMZM}(t) & \propto \left[J_{0}(m_{1})+J_{0}(m_{2})-J_{0}(m_{3})\right]J_{1}(\alpha )\left\{\begin{array}{l} \exp\left[j2\pi (f_{c}+f_{0}+kt)t\right]\\ +\exp\left[j2\pi (f_{c}-f_{0}-kt)t\right] \end{array}\right\}\\ & +J_{1}(m_{1})J_{1}(\alpha )\left\{\begin{array}{l} \exp\left[j2\pi (f_{c}-f_{0}-kt+2k\tau )t\right]\\ +\exp\left[j2\pi (f_{c}-3f_{0}-3kt+2k\tau )t\right]\\ +\exp\left[j2\pi (f_{c}+3f_{0}+3kt-2k\tau )t\right]\\ +\exp\left[j2\pi (f_{c}+f_{0}+kt-2k\tau )t\right] \end{array}\right\} \end{array}$$

An optical bandpass filter (OBPF) is used to select the signals related to $f_{0}+f_{c}+kt$ and $f_{0}+f_{c}+kt-2k\tau$, and then the IF signal with the target distance information can be obtained by a photodetector,

(14) $$L=\frac{c.\tau }{2}=\frac{c}{2}.\frac{∆f}{2k}=\frac{c.T.∆f}{4B}$$

Overall, the proposed system can simultaneously achieve frequency-doubled linear frequency modulation signal generation, de-chirp reception, self-interference cancellation, and frequency measurement in an integrated transmit-receive radar.

## 3. Results

A proof-of-concept experiment based on the setup shown in Figure 1a is carried out. CW light with a wavelength of 1550.12 nm and a power of 14 dBm from a laser source (NKT Photonics, Koheras BASIK, Beijing, China) is sent to MZM1 (Fujitsu FTM7928, Beijing, China). Moreover, an LFM signal with a temporal period of 60 μs and a frequency range of 6–9 GHz generated by a signal generator (LMS-183CX, Beijing, China) is injected into MZM1, which biases at the minimum transmission point to obtain the CS-DSB signal. An optical coupler (OC, Conquer, KG-OC-1550-P-3-2, Beijing, China) is used to split the output of MZM1 into three parts. One part of the OC is sent to MZM2 (Fujitsu FTM7928, Beijing, China) as the optical carrier for frequency measurement, while MZM2 is driven by the SUT from the receiving antenna. Two electrical IF filters with different center frequencies are used after PD1 (Conquer, KG-PR200M-A-FC, Beijing, China) to select the IF signal. After that, the two selected IF signals are digitalized by a real-time oscilloscope and processed by a computer. The other part is used as the carrier of the DPMZM (Fujitsu FTM7962EP, Beijing, China) for de-chirp reception in an integrated transmit-receive radar.

The last part of the OC is input into PD2 (Conquer, KG-PD20G-A-FC, Beijing, China) to generate a frequency-doubled LFM signal as the transmit signal for de-chirp reception in an integrated transmit-receive radar. The PD2 output electrical signal is divided into two branches with a 3 dB electrical coupler (Marki, PD-0440, Beijing, China). One branch is amplified by an EA as the transmit signal and input into the transmitting antenna as the transmit signal. The other is sent to an ETDL (ATM, PNR P1409A, Beijing, China) and an EATT (Narda, 4796, Beijing, China) as a reference signal for transmitting signal interference cancellation and loaded onto one sub-MZM of the DPMZM. For the LFM radar receiver, the signals received from the antenna, including the echo signal and the self-interference signal, are both amplified by the LNA and then loaded onto the other sub-MZM of the DPMZM. By matching the power and delay of the reference signal and changing the main bias voltage of the DPMZM, the interference signal can be canceled in the optical domain. After the DPMZM, an OBPF (OTF-980, Beijing, China) is applied to filter out the unwanted sidebands and then injected into PD3 to perform the photoelectrical conversion. Finally, the de-chirp receiving signal without self-interference is obtained.

**Figure 3:**
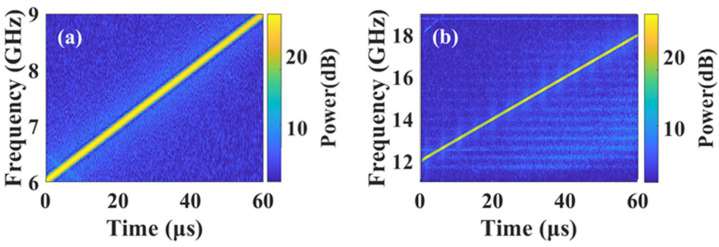
Corresponding instantaneous frequency of the transmit LFM signal in one period (60 μs). (**a**) The input chirp signal, (**b**) the output chirp signal.

The function of frequency-doubled transmit signal generation is studied. An oscilloscope (OSC, Tektronix DPO75902SX, Beijing, China) is used to capture the waveform of the original signal and the frequency-doubled signal. The corresponding time-frequency characteristics are shown in Figure 3a and Figure 3b, respectively. The center frequency and instantaneous bandwidth of the frequency-doubled LFM signal are 15 GHz and 6 GHz (12–18 GHz), both of which are doubled, and the signal has good linear characteristics.

**Figure 4:**
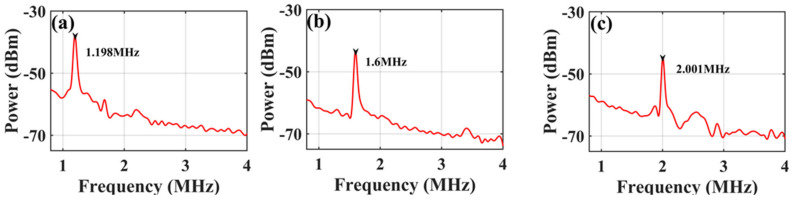
The output spectrum when the length of the electrical cable is (**a**) 1 m, (**b**) 2 m and (**c**) 3 m.

To verify the system’s ability to perform de-chirp reception, electrical cables with calibrated lengths of 0.8 m, 1.6 m, and 2.4 m are selected to simulate the distance of targets at three different positions. The electrical cables are connected directly to the transmitting and receiving ports in the system, and the IF spectra of the receiver are shown in Figure 4a–c. The distance calculated above with Equation (14) is a one-way distance obtained from the bidirectional microwave transmission time. Therefore, when electrical cables are used for equivalent verification in this paper, the cable length should be twice the distance calculated by Equation (14). In addition, there is an inherent length of the fiber optic link between the transmitting and receiving ports, which is measured to be 1.601 m through the vector network. Therefore, the calculation length of the test cable also needs to subtract the inherent length of the system. Based on the measured IF frequency values, three different lengths of electrical cables can be calculated: 0.795 m, 1.599 m, and 2.401 m. The error between the distance tested and the actual length is within 0.5 cm.

**Figure 5:**
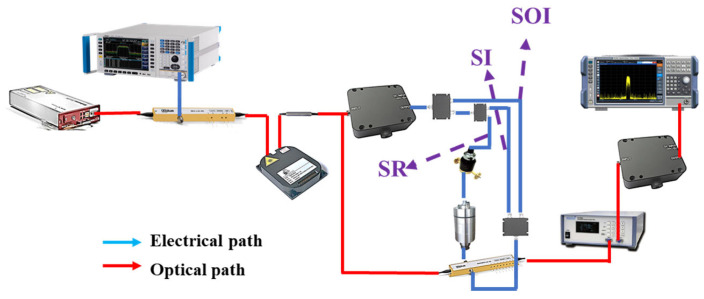
Schematic diagram for simulation of target echo signals at different distances with/without interference.

**Figure 6:**
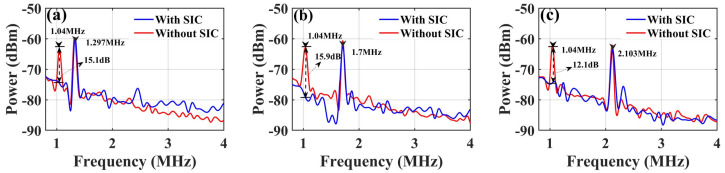
The output spectrum with or without interference cancellation when the length of the electrical cable is (**a**) 1 m, (**b**) 2 m, and (**c**) 3 m.

Second, different calibrated lengths of electrical cables were also used to simulate and verify target position measurements with or without self-interference signals. The testing scheme is shown in Figure 5. A 3 dB electrical coupler splits the output of the EA into two parts. One is launched into an electrical cable with a length of 0.4 m as the transmitting self-interference signal, and the other connects cables of different lengths to be tested. In the receiving port, the self-interference signal cable and the test cable are combined by another 3 dB electrical coupler and then input to the RF port of the DPMZM to complete the de-chirp receiver. The red curve shows the de-chirped reception IF signal without self-interference cancellation in Figure 6a–c. It is obvious that there is a de-chirped self-interference IF signal with a frequency of 1.04 MHz on the spectrum, which is caused by the equivalent verification test cable. 

Similarly, the self-interference cable length can be calculated as 0.407 m, which includes the lengths introduced by two 3 dB couplers. By adjusting the ETDL, EATT, and main bias voltage of the DPMZM, the de-chirped reception IF signal with interference cancellation is shown as a blue curve in Figure 6a–c. The power of the de-chirped IF signal generated by the interference signal is well suppressed, and the corresponding cancellation depths are 15.1 dB, 15.9 dB, and 12.1 dB. The simulated cable lengths were calculated to be 0.993 m, 1.799 m, and 2.605 m, respectively. It should be noted that the length error of the tested cable between here and above is caused by the addition of a 3 dB coupler and connecting lines during testing.

**Figure 7:**
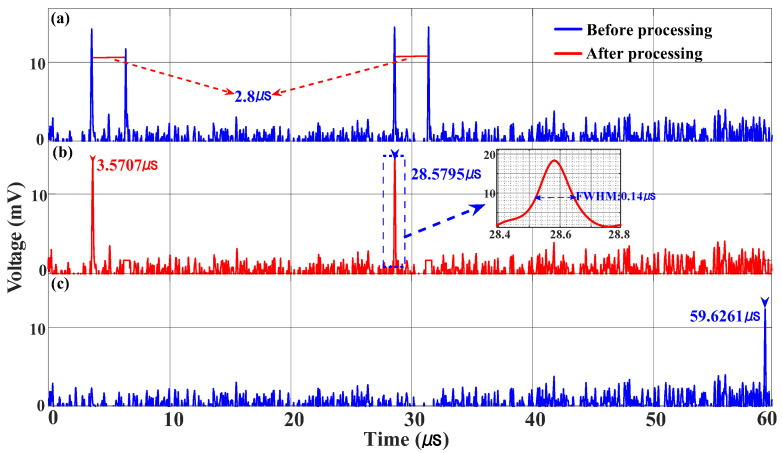
Waveform acquired by the ADC when a single-frequency signal with a frequency of (**a**,**b**) 15 GHz, (**c**) 18.1 GHz is under test.

To demonstrate the feasibility of the frequency measurement function, an IF filter (GBPF140-13M-SMF, Beijing, China) with a center frequency of 140 MHz was selected for the experiment, and the frequency range of the LFM signal used was from 6 GHz to 9 GHz. After optical domain doubling, the obtained LFM signal frequency range is 12 GHz–18 GHz. Therefore, it can measure the frequency of the SUT at 12.14–18.14 GHz. The time required for a single measurement is determined by the duration of the swept LFM signal; the higher the repetition rate of LFM, the faster the measurement speed. The measurement time for a single experiment is 60 μs. In the experiment, the SUT signal is modulated to MZM2, which is biased at the quadrature point. The SUT signals with frequencies of 12.5 GHz and 15 GHz are combined and then sent to MZM2. The time pulses after the IF filter are collected by an oscilloscope with a sampling rate of 1 GSa/s, and the time pulses are shown in Figure 7a. With the data processing method mentioned in Part 2, pulse pair identification was completed, and a one-to-one time-frequency mapping was obtained, as shown in Figure 7b. By measuring the time of the pulses and introducing them into Equation (6), the SUT signal frequencies are estimated to be 12.49707 GHz and 14.9979 GHz. Then, the time pulse waveforms for frequencies greater than 18 GHz are shown in Figure 7c. With the data processing methods mentioned in Part 2, the time pulse signal has been identified and measured. The estimated frequency of the tested signal was calculated to be 18.1026 GHz. This is close to the actual SUT signal and it also proves that the proposed system has the ability to measure multiple frequency signals.

**Figure 8:**
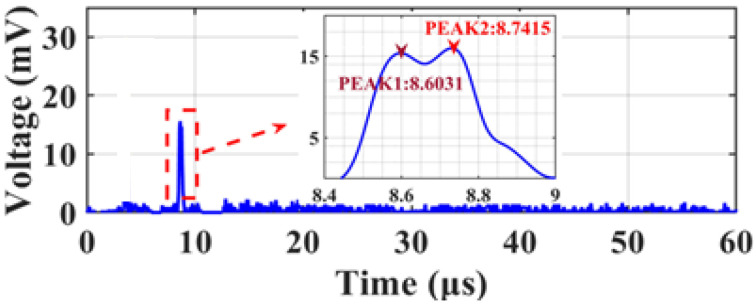
Waveform acquired by the OSC when a two-tone signal has a frequency spacing of 14 MHz.

The resolution of the frequency measurements was investigated. When testing a single-frequency signal, the full width at half maximum (FWHM) of the IF pulse is approximately 0.14 μs, as shown in the illustration of Figure 7b. According to the mapping relationship between frequency and time, a duration of 0.14 μs corresponds to a frequency interval of 14 MHz, which means that the frequency measurement resolution is 14 MHz. In the validation experiment, a two-tone signal (13.00 GHz and 13.014 GHz) with a frequency interval of 14 MHz was used as the SUT, and the results are shown in Figure 8, where the two pulses can still be distinguished, which shows that a measurement resolution of 14 MHz is achieved.

**Figure 9:**
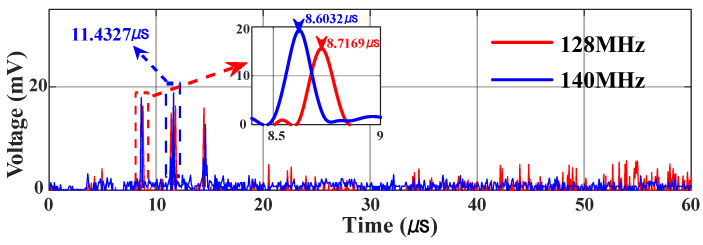
Waveform acquired by the OSC when a two-tone signal has a frequency difference of 280 MHz under test with different center frequencies of the IF filter.

The center frequency of the IF filter used in the experiment is 140 MHz, so there is a fixed time difference of 2.8 μs between the time pulse pairs after the IF filter, which maps to a frequency difference of 280 MHz in the frequency domain. The time pulse pairs without data processing and their time relationships are shown in Figure 7a. When measuring two-tone signals with a frequency difference of 280 MHz, the symmetrical pulse pairs introduced by the IF filter cause the later time pulse of the previous frequency to overlap with the earlier pulse of the next frequency, which interferes with the data processing of the two frequency measurements. Therefore, this may lead to the loss of the frequency of the signal to be tested with a single filter. In the proposed system, the time difference between the two time-pulses generated by the frequency-time mapping of the frequency to be measured is related to the center frequency of the IF filter. Therefore, two IF filters with different center frequencies can be used to avoid the loss of frequency measurement with a frequency difference of twice the center frequency of the IF filter. In the verification experiment, the microwave frequencies tested were 13.00 GHz and 13.28 GHz. Using a filter with a center frequency of 140 MHz and completing the time pulse acquisition, the resulting time-pulse graph is shown as the blue curve in Figure 9. It contains three time-pulses, and the time difference between adjacent pulses is 2.8 μs. It is impossible to distinguish the time pulses directly according to the method in Part 2. Therefore, another IF filter data processing channel with a center frequency of 128 MHz is added. The time-pulse graph is shown as the red curve in Figure 9, which includes two sets of pulse pairs with a time difference of approximately 2.56 μs. The method described in Part 2 can be used to distinguish the time pulses and process them into a one-to-one mapping between frequency and time. It can be seen from the illustration in Figure 9 that the two data acquisition channels have a time difference of approximately 0.12 μs between the first pulse of the pulse pair, corresponding to a frequency difference of approximately 12 MHz, which is the center frequency difference of the two IF filters. This indicates that the proposed system can solve the ambiguity in frequency measurements.

**Figure 10:**
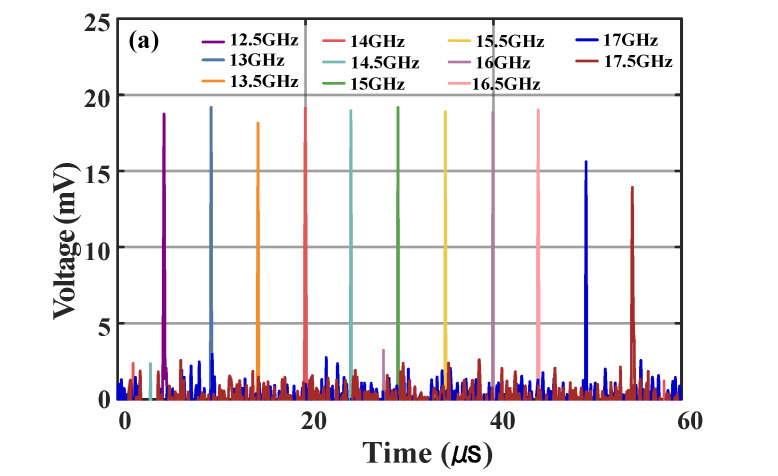

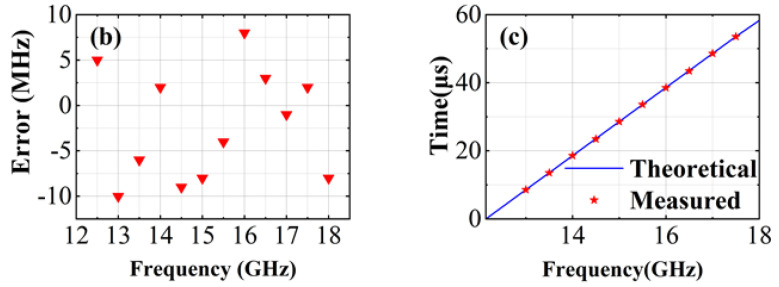
(**a**) Pulse waveforms after the IF filter for SUT signal frequencies ranging from 12.5 to 17.5 GHz with a frequency step of 0.5 GHz, (**b**) the measurement errors versus the input frequencies, and (**c**) the measured and theoretical time positions of the pulses.

Furthermore, to verify the frequency range of the designed system, frequencies ranging from 12.5 to 17.5 GHz with a frequency step of 0.5 GHz are tested, and the results are shown in Figure 10. The waveforms in Figure 10b show the measurement error at different frequencies, and the measurement error is less than ±11 MHz. The measured and theoretical time positions of the pulse are shown in Figure 10c.

Owing to the broadband, flexible, and reconfigurable characteristics of microwave photonics technology, multiple electro-optical modulators, optical filters, optical couplers, and other optical components are connected to enable multi-function microwave signal processing functions in a single microwave photonics system. The limitations of the operating frequency range for the proposed photonic-based multifunctional system in this article, which is used to integrate transmit-receive radar systems, mainly include the following two aspects. On the one hand, owing to electronic bottlenecks, the external LFM signal generator has a limited scanning bandwidth. On the other hand, while the operating frequency of the modulator in the frequency-doubled linear frequency modulation signal generation is half of the system’s highest frequency, the PD in the frequency-doubled linear frequency modulation signal generation and the electro-optical modulator in the frequency measurement and de-chirp reception need to cover the system’s highest operating frequency. Thus, the operating bandwidths of frequency-related devices, such as those with MZM and DPMZM modulators and PDs, also limit the operating frequency range of the proposed system. Owing to the limited bandwidth of the electronic LFM signal generators, optoelectronic oscillators can be used to generate high-frequency and broadband LFM signals [31,32,33]. By establishing a photoelectric oscillation loop in the proposed scheme, the use of external electrical local oscillator signal sources can be eliminated, and the ability of all-optical signal generation and processing can be increased. For frequency-dependent devices, the frequency range of commercial PD exceeds 100 GHz, and the MZM and DPMZM based on LNOI also exceed 110 GHz [34,35,36]. By replacing suitable high-frequency devices, the proposed scheme can be extended in its operating frequency range to meet the requirements of current typical miniaturized broadband radar signal processing systems. In addition, the MZM and DPMZM used in the experiment were both manufactured based on lithium niobate material, which also provides the possibility of integrating them into a thin film of lithium niobate in the future. Therefore, in the future, we can design and manufacture an integrated device on thin-film lithium niobate, which has the characteristics of a compact structure and wide operating frequency range, making it suitable for high-frequency and broadband miniaturized integrated multifunctional radar signal processing systems. Additionally, the LNOI integrated device and the chip-scale LFM signal generator based on an optoelectronic oscillator provide the possibility of all-optical monolithic integration for a multifunctional radar signal processing system.

## 4. Conclusions

In conclusion, we have proposed and demonstrated a photonics-based multifunctional system for integrated transmit-receive radar systems. The proposed MWP system can simultaneously achieve frequency-doubled linear frequency modulation signal generation, de-chirp reception, self-interference cancellation, and frequency measurement. The experiment results of the proposed scheme show that an LFM signal with a frequency range of 12–18 GHz was obtained with photonic frequency doubling. The photonic-assisted self-interference cancellation can reduce the impact of interference signals in radar de-chirp reception by more than 12.1 dB for an LFM signal bandwidth of 6 GHz, and a measurement resolution better than 14 MHz is achieved in the range of 12.14 GHz to 18.14 GHz. The shared frequency doubled the LO signal between the frequency measurement system and the radar signal processing system, increasing the operating bandwidth and center frequency. Combining multiple functions in a microwave photonic link can greatly simplify the system structure and help reduce system costs. The experimental results demonstrate the advantages of the proposed system and provide possible solutions for future electrical systems with multiple functions.

## Data Availability

The original contributions presented in the study are included in the article, further inquiries can be directed to the corresponding author.
